# Prevalence of tobacco consumption and the associated factors among the adults in an urban slum: Findings from the WHO STEPwise survey

**DOI:** 10.18332/tid/154636

**Published:** 2022-10-31

**Authors:** Shama Razzaq, Muhammad Luqman Farrukh Nagi, Unsa Athar, Tahseen Kazmi, Thamer Alslamah, Sana Naz, Adil Abalkhail

**Affiliations:** 1 Division of Environment and Sustainability, The Hong Kong University of Science and Technology, Hong Kong, People's Republic of China; 2 Department of Community Medicine, Shalamar Medical and Dental College, Lahore, Pakistan; 3 Department of Community Medicine, Central Park Medical College, Lahore, Pakistan; 4 Department of Public Health, College of Public Health and Health Informatics, Qassim University, Al Bukairiyah, Saudi Arabia

**Keywords:** tobacco consumption, adult population, risk factors, urban slum

## Abstract

**INTRODUCTION:**

Despite Pakistan’s stringent tobacco control policy, its effective implementation has always been a challenge, leading to rising tobacco consumption. The aim of the study is to determine the prevalence of tobacco consumption and associated risk factors in the adult population of deprived urban areas.

**METHODS:**

A community-based, cross-sectional survey was conducted from February to July 2019, comprising 607 adults residing in the urban squatter settlement of Lahore using a standardized questionnaire, the WHO STEPwise approach. The outcome was current use of tobacco and/or smokeless tobacco daily. Multivariable logistic regression was applied to determine factors associated with smoking and smokeless tobacco consumption.

**RESULTS:**

Among 607 participants, about 64% were females, 49.3% were illiterate, 64.9% were currently unemployed, and 47.1% belonged to the low-income group. The prevalence of tobacco smoke was 10.5% (95% CI: 8.07–12.93), and smokeless tobacco consumption was 8.6% (95% CI: 6.38–10.82). Multivariable logistic regression found that smokeless tobacco was more likely among the aged 50–59 years (AOR=4.1; 95% CI: 1.1–13.8) and unemployed (AOR=3.6; 95% CI: 1.1–12.2). Whereas tobacco smoking was more likely among the aged 30–39 years (AOR=5.5; 95% CI: 1.8–16.7), Urdu ethnicity (AOR=2.9; 95% CI: 1.2–7.3), unemployed (AOR=6.6; 95% CI: 2.9–14.9), and never exposed to any media (AOR=3.2; 95% CI: 1.8–17.4). Participants exposed to health warnings were less likely to smoke (AOR=0.02; 95% CI: 0.01–0.05).

**CONCLUSIONS:**

This study reports a high prevalence of tobacco consumption among adults and calls for effective policy implementation using a multi-pronged approach, including health professionals and media, to spread awareness about the harmful effects of tobacco and endorsement of health warnings on tobacco packaging.

## INTRODUCTION

Tobacco consumption is an important risk factor and responsible for increasing non-communicable diseases (NCDs), but a preventable cause of mortality and morbidity^1^. The World Health Organization (WHO) reported that the prevalence of smoking globally among adults was 19.2%, to which over 8 million deaths have been attributed, and is responsible for about 150 million disability-adjusted life years (DALYs) due to smoking-associated illnesses each year^1,2^. Furthermore, the burden of smokeless tobacco consumption has been increasing, with consumption of smokeless tobacco in 115 countries accounting for 1.7 million DALYS lost and approximately 60000 deaths due to mouth, pharynx, and throat cancers, with South-East Asia accounting for over 85% of this burden^3^.

Despite the lack of timely and accurate data, the rising trend we observed in Pakistan for the prevalence of tobacco use was approximately 20% in 2003, which now has risen to 45.5%, reported in a nationwide household survey published in 2019^4^. As mentioned, tobacco consumption is on the rise in Pakistan. Tobacco is consumed not only in smoked forms (cigarettes and waterpipes) but also in smokeless forms (naswar, gutka, and chewing paan)^5^. The evidence from the household survey further mentioned that out of 45.5% of tobacco consumers, the prevalence of only tobacco smokers was 21.2%, whereas 18.5% were only smokeless tobacco users and 5.8% were users of both smoke and smokeless tobacco^4^. Considering the heavy toll on human health due to the harmful effects of smoking, Pakistan needs to implement strict measures. Therefore, Pakistan became a signatory to the World Health Organization (WHO) Framework Convention on Tobacco Control (FCTC)^6^. The FCTC provides support to countries for implementing tobacco control programs comprehensively through the MPOWER approach to prevent people from tobacco use and exposure, monitor tobacco use and interventions, protect people, offer help, warn about the dangers, enforce bans, and raise taxes^7^.

Pakistan, however, is lagging behind in the effective implementation of MPOWER due to several gaps in existing policies as it is limited to raising taxes and warnings for cigarettes only and ignoring other tobacco products. Also, ‘offering help to quit tobacco’ is not catered by laws, as well as there is a sub-optimal implementation of legislations in terms of enforcing bans and promotions of the products and monitoring^8^. Hence, there is dire need to identify the factors related to tobacco consumption post-implementation of FCTC to address uncontrolled tobacco consumption effectively. Another important element of a dearth in studies regarding tobacco consumption (and other risk factors for NCDs) among the urban slum population, is fulfilling the health needs of the poorest urban communities, as a study highlighted the substantial intra-urban disparities in tobacco use^9^. The exclusion of vulnerable populations from the research will create a roadblock in strategizing for the reduction of non-communicable diseases and achieving global health^10^. Hence, our survey covered deprived populations who belonged to the urban squatter settlement of Lahore in order to explore inequalities in the risk of non-communicable diseases and associated risk factors. We conducted a survey using the WHO STEPwise approach, which aimed for comprehensive surveillance of NCD risk factors, and this study focuses on tobacco use and exposure^11^.

Therefore, the objective of the study was to determine the prevalence of tobacco consumption and associated risk factors at different levels among the adult population of deprived urban areas in Lahore, Pakistan.

## METHODS

### Study design and setting

This population-based cross-sectional survey was conducted in Lahore, Pakistan from February to July 2019. A two-stage cluster sampling technique was used. In the first stage, Union Councils (UCs) were selected from the Gulberg and Shalamar town areas in Lahore, Pakistan. People living in these two areas of Lahore had an average income below the per capita income of Pakistan in 2019. In the second stage, a random sampling method was applied to consecutively recruit 607 participants aged 30–69 years both males and females, residing in the urban squatter settlement of Union Councils 120 and 122 of Lahore, and the UCs were selected on convenience based on logistic support.

### Participant recruitment

The area survey of both the union councils 120 and 122, as selected clusters, and mapping of the households was done after acquiring permission from the local authorities of the respective union councils. In both clusters (primary sampling unit), approximately 50000 people live, of which 9000 to 10000 are adults. Line listing was carried out within both clusters where a total of 400 households (secondary sampling units) from both UCs were visited to identify eligible participants and mark them with an identification number. All eligible participants (residents of these union councils for at least 6 months) from the identified households were recruited. Participants were ineligible if they refused to provide consent, had severe comorbid conditions with a life expectancy of less than 1 year, or had other serious conditions which could interfere in participation or inability to complete the study (e.g. terminal stage of cancer, HIV, TB), or were temporary residents of these UCs.

### Sample size

The minimum sample size was calculated to be 530 (using the Open Epi Version 3.01) based on the prevalence of tobacco consumption of 14.5% from a population-based survey^12^, keeping a margin of error 3%, a confidence level of 95%, and a 1.5 design effect. Then, we inflated the sample size by approximately 15% to adjust for non-response and refusal, hence the final sample size was 610.

### Interview procedure and study variables

Interviews were carried out by a trained field team in the Urdu language using a structured questionnaire, i.e. the WHO STEPwise Approach to Chronic Disease Risk Factor Surveillance (STEPS) questionnaire, which was adapted and used in this survey to identify the prevalence of tobacco use and its possible associated risk factors^11^. It included questions about socioeconomic and demographic variables (age, gender, ethnicity, education level, income, and occupational status); behavioral (physical activity, healthy lifestyle adoption, and health checkups) and metabolic (having hypertension, diabetes, hyperlipidemia, and a family history of chronic diseases) risk factors; and other factors, such as exposure to environmental tobacco smoke. Intense physical activity was defined as ‘vigorous-intensity activity for at least 10 minutes continuously that causes large increases in breathing or heart rate, such as carrying or lifting heavy loads, digging or construction work’; moderate physical activity was defined as ‘moderate-intensity activity for at least 10 minutes continuously that causes small increases in breathing or heart rate like brisk walking’; and mild physical activity was defined as ‘a walk for 10 minutes at least for travelling during a week’. The WHO STEPS questionnaire of the WHO STEPwise approach to non-communicable disease risk factor surveillance was used for operational definitions of all included variables^11^. Body mass index (BMI) categories were developed according to WHO criteria widely used in studies, such as: normal weight (18.5–24.9 kg/m^2^), overweight (25–29.9 kg/m^2^), and obese (30 kg/m^2^)^13^. The outcome variable of interest in this study was the ‘current use of smoke and smokeless tobacco on a daily basis’^11^.

### Anthropometric and biochemical measurements

Anthropometric measurements were recorded including height and weight to calculate the body mass index. Assessment of random blood sugar, cholesterol level, and systolic and diastolic blood pressure, and were performed according to standardized methods as per the WHO STEPS protocol^11^, to ensure consistency of recordings with reliable equipment. Variability was minimized by standardized training of all survey teams to use STEPS questionnaire and record blood pressure, random blood sugar and other measurements.

### Statistical analysis

Data were analyzed using SPSS version 19.0 Frequencies and percentages were calculated for categorical variables. Chi-squared test was done to assess the sample distribution according to tobacco consumption status. Univariable logistic regression was carried out to calculate unadjusted odds ratios of determinants associated with consumption of smoke and smokeless tobacco, separately. Multivariable logistic regression analysis was done to estimate the adjusted odds ratios for associated factors of consumption of smoke and smokeless tobacco, separately.

## RESULTS

A total of 610 adults aged ≥30 years were contacted to participate in the selected households, out of which 607 agreed to participate. The non-responders were very few, this could be due to the sensitivity attached to tobacco smoking queries, and were reluctant to provide any information.

### Sociodemographic and economic characteristics

Of the participants, 39.7% were aged 30–39 years. The majority of the participants were females (64.4%) and the most common ethnicity among the study participants was Urdu (48.3%). Approximately half of the participants (49.3%) were illiterate, the majority (64.9%) of the participants were currently unemployed and about half (47.1%) belonged to the low-income group. A total of 126 (20.8%) participants reported exposure to environmental tobacco smoke (Table 1).

**Table 1:** Characteristics related to sociodemographics, exposure related to awareness, life style adopted, health-related issues, anthropometric details, and family history of disease, among adults (N=607)

*Characteristics*	*n (%)*
**Sociodemographic**	
**Age** (years)	
30–39	241 (39.7)
40–49	147 (24.2)
50–59	125 (20.6)
≥60	94 (15.5)
**Gender**	
Male	216 (35.6)
Female	391 (64.4)
**Marital status**	
Never married	22 (3.6)
Ever married	585 (96.4)
**Ethnicity**	
Urdu	293 (48.3)
Punjabi	229 (37.7)
Pushto	85 (14.0)
**Education level**	
Intermediate and above	56 (9.2)
Up to Secondary	252 (41.5)
Illiterate	299 (49.3)
**Socioeconomic status**	
High income	154 (25.4)
Middle income	167 (27.5)
Low income	286 (47.1)
**Occupation**	
Employed	213 (35.1)
Unemployed	394 (64.9)
**Exposure to environmental tobacco smoke**	
Yes	126 (20.8)
No	481 (79.2)
**Exposure related to awareness**	
**Media**	
None	154 (25.4)
At least one source	249 (41.0)
All three sources	204 (33.6)
**Health warnings**	
Yes	119 (19.6)
No	488 (80.4)
**Life style adopted**	
**Intense physical activity**	
Yes	150 (24.7)
No	457 (75.3)
**Moderate physical activity**	
Yes	147 (24.2)
No	460 (75.8)
**Mild physical activity**	
Yes	284 (46.8)
No	323 (53.2)
**Ever BP monitoring**	
Yes	281 (46.3)
No	326 (53.7)
**Ever random blood sugar monitoring**	
Yes	201 (33.1)
No	406 (66.9)
**Ever cholesterol monitoring**	
Yes	81 (13.3)
No	526 (86.7)
**Ever advised for quitting tobacco use**	
Yes	187 (30.8)
No	420 (69.2)
**Health-related issues**	
**Hypertension**	
Yes	325 (53.5)
No	282 (46.5)
**Diabetes mellitus**	
Yes	190 (31.3)
No	417 (68.7)
**Hyperlipidemia**	
Yes	69 (11.4)
No	538 (88.6)
**BMI** (kg/m^2^)	
Normal weight	190 (31.3)
Overweight	208 (34.3)
Obese	209 (34.4)
**Family history of chronic diseases**	
**Stroke**	
Yes	50 (8.2)
No	557 (91.8)
**IHD**	
Yes	110 (18.1)
No	497 (81.9)
**Hypertension**	
Yes	234 (38.6)
No	373 (61.4)
**Diabetes**	
Yes	254 (41.8)
No	353 (58.2)

BMI: body mass index.

### Exposure related to awareness

Of the total participants, 41% were exposed to at least one media source regarding awareness about harmful effects of tobacco use while only 19.6% reported exposure to health warnings on tobacco product packaging (Table 1).

### Healthy lifestyle adopted

Mild, moderate and intense physical activity was reported by 46.8%, 24.2% and 24.7%, respectively. Ever monitoring of blood pressure (BP), random blood sugar (RBS), and cholesterol was reported by 46.3%, 33.1% and 13.3%, respectively, while only 30.8% of ever tobacco users reported being advised by a healthcare professional for quitting tobacco use (Table 1).

### Health-related issues

The majority (53.5%) of the participants had hypertension in our study, while about one-third (31.3%) of the participants had diabetes and only 11.4% reported having a doctor diagnosis of hyperlipidemia. Overweight were 34.3% and obese were 34.4% in our study (Table 1).

### Family history of chronic diseases

Of the total participants, 41.8%, 38.6%, 18.1% and 8.2% reported family history of diabetes, hypertension, ischemic heart disease (IHD), and stroke, respectively (Table 1).

The prevalence of current tobacco smoke consumption was 10.5% while prevalence of smokeless tobacco consumption was 8.6%. Further details are given in the Supplementary file.

Univariable logistic regression showed that current tobacco smoking was more likely among those aged 30–39 years (OR=2.1; 95% CI: 1.1–4.5), the Urdu ethnicity (OR=2.7; 95% CI: 1.4–5.3), the unemployed (OR=7.4, 95% CI: 4.2–13.8), never exposed to any media (OR=2.1; 95% CI: 1.1–4.2), having no intense physical activity (OR=2.4; 95% CI: 1.4–4.2), having hypertension (OR=2.6; 95% CI: 1.5–4.5), having diabetes mellitus (OR=1.8; 95% CI: 1.1–3.5), and that were obese (OR=2.7; 95% CI: 1.3–5.2).

Participants who were less likely to consume smoked tobacco were exposed to passive smoking (OR=0.2; 95% CI: 0.1–0.3), exposed to health warning on packaging (OR=0.4; 95% CI: 0.02–0.07), and were ever advised for quitting tobacco use (OR=0.4; 95% CI: 0.2–0.7). Current smokeless tobacco consumers were more likely among the unemployed (OR=5.3; 95% CI: 2.8–9.9), never exposed to any media (OR=3.9; 95% CI: 1.5–9.7), having no intense physical activity (OR=2.2; 95% CI: 1.3–4.3), having diabetes mellitus (OR=2.6; 95% CI: 1.2–5.8), and were obese (OR=2.1; 95% CI: 1.0–4.2). Participants who were less likely to consume smokeless tobacco were exposed to passive smoking (OR=0.1; 95% CI: 0.03–0.13), exposed to health warnings on packaging (OR=0.3; 95% CI: 0.2–0.5), and were ever advised for quitting tobacco use (OR=0.5; 95% CI: 0.3–0.9) (Table 2).

**Table 2:** Univariable logistic regression analysis for factors associated with tobacco consumption among adults (N=607)

*Characteristics*	*Current smoking tobacco*		*Current smokeless tobacco*	
	*AOR (95% CI)*	*p*	*AOR (95% CI)*	*p*
**Age** (years)				
30–39	2.1 (1.1–4.5)	0.04*	0.8 (0.3–1.8)	0.61
40–49	1.1 (0.5–2.3)	0.77	1.0 (0.4–2.6)	0.92
50–59	1.6 (0.7–3.7)	0.23	1.5 (0.5–4.4)	0.42
≥60 (Ref.)	1		1	
**Gender**				
Male	0.07 (0.03–1.5)	0.47	0.1 (0.07–1.2)	0.21
Female (Ref.)	1		1	
**Marital status**				
Never married (Ref.)	1		1	
Ever married	2.6 (0.9–7.3)	0.06	2.4 (0.8–7.6)	0.11
**Ethnicity**				
Punjabi (Ref.)	1		1	
Urdu	2.7 (1.4–5.3)	0.00*	0.9 (0.4–1.8)	0.88
Pushto	1.4 (0.6–3.0)	0.37	0.4 (0.2–1.0)	0.06
**Education level**				
≥Intermediate (Ref.)	1		1	
Up to Secondary	1.4 (0.6–3.3)	0.39	0.5 (0.2–1.9)	0.36
Illiterate	1.5 (0.6–3.4)	0.34	0.5 (0.1–2.0)	0.41
**Socioeconomic status**				
High income (Ref.)	1		1	
Middle income	1.2 (0.5–2.3)	0.66	0.7 (0.3–1.6)	0.41
Low income	1.2 (0.6–2.1)	0.61	0.6 (0.3–1.4)	0.29
**Occupation**				
Employed (Ref.)	1		1	
Unemployed	7.4 (4.2–13.8)	0.00*	5.3 (2.8–9.9)	0.00*
**Exposure to environmental tobacco smoke**				
Yes	0.2 (0.1–0.3)	0.00*	0.1 (0.03–0.13)	0.00*
No (Ref.)	1		1	
**Exposure to media**				
None	2.1 (1.1–4.2)	0.03*	3.9 (1.5–9.7)	0.00*
At least one	1.9 (1.1–3.5)	0.02*	2.0 (1.1–3.8)	0.02*
All three (Ref.)	1		1	
**Exposure to health warnings**				
Yes	0.4 (0.02–0.07)	0.00*	0.3 (0.2–0.5)	0.00*
No (Ref.)	1		1	
**Intense physical activity**				
Yes (Ref.)	1		1	
No	2.4 (1.4–4.2)	0.00*	2.4 (1.3–4.3)	0.00*
**Moderate physical activity**				
Yes (Ref.)	1		1	
No	1.2 (0.7–2.2)	0.44	1.1 (0.5–2.0)	0.89
**Mild physical activity**				
Yes (Ref.)	1		1	
No	1.3 (0.7–2.2)	0.28	1.0 (0.5–1.5)	0.69
**Having hypertension**				
Yes	2.6 (1.5–4.5)	0.00*	1.6 (0.9–2.9)	0.09
No (Ref.)	1		1	
**Having diabetes mellitus**				
Yes	1.8 (1.1–3.5)	0.04*	2.6 (1.2–5.8)	0.01*
No (Ref.)	1		1	
**Having hyperlipidemia**				
Yes	2.8 (0.8–9.2)	0.08	1.2 (0.4–3.1)	0.67
No (Ref.)	1		1	
**Ever advised for quitting tobacco use**				
Yes	0.4 (0.2–0.8)	0.00*	0.5 (0.3–0.9)	0.03*
No (Ref.)	1		1	
**BMI** (kg/m^2^)				
Normal weight (Ref.)	1		1	
Overweight	1.9 (1.1–3.5)	0.03*	1.6 (0.8–3.2)	0.14
Obese	2.7 (1.3–5.2)	0.00*	2.1 (1.0–4.2)	0.04*
**Family history of stroke**				
Yes	1.9 (0.5–6.3)	0.28	0.5 (0.2–1.2)	0.15
No (Ref.)	1		1	
**Family history of IHD**				
Yes	0.7 (0.4–1.4)	0.41	1.2 (0.5–2.7)	0.59
No (Ref.)	1		1	
**Family history of hypertension**				
Yes	1.3 (0.7–2.2)	0.32	0.6 (0.3–1.2)	0.14
No (Ref.)	1		1	
**Family history of diabetes**				
Yes	1.1 (0.6–1.7)	0.83	1.3 (0.7–2.5)	0.27
No (Ref.)	1		1	

BMI: body mass index.

* Significant at p≤0.05.

Multivariable logistic regression analysis found that current tobacco smokers were more likely among those aged 30–39 years (AOR=5.5; 95% CI: 1.8–16.7) and 50–59 years (AOR= 5.6; 95% CI: 1.6–19), the Urdu ethnicity (AOR=2.9; 95% CI: 1.2–7.3), the unemployed (AOR=6.6; 95% CI: 2.9–14.9), and never exposed to any media (AOR=3.2; 95% CI: 1.8–17.4). Participants who were less likely to consume tobacco smoke currently were exposed to passive smoking (AOR= 0.3; 95% CI: 0.1–0.5), and exposed to health warnings on packaging (AOR=0.02; 95% CI: 0.01–0.05). For smokeless tobacco consumers, multivariable logistic regression analysis found that it was more likely among those aged 50–59 years (AOR=4.1; 95% CI: 1.1–13.8), and the unemployed (AOR=3.6; 95% CI: 1.1–12.2). Participants who were less likely to use smokeless tobacco currently were less exposed to passive smoking (AOR=0.1; 95% CI: 0.03–0.2) (Table 3).

**Table 3:** Multivariable logistic regression analysis for factors associated with tobacco consumption among adults (N=607)

*Characteristics*	*Current smoking tobacco*		*Current smokeless tobacco*	
	*AOR (95% CI)*	*p*	*AOR (95% CI)*	*p*
**Age** (years)				
30–39	5.5 (1.8–16.7)	0.00*	1.4 (0.5–3.9)	0.48
40–49	1.9 (0.6–5.6)	0.22	1.5 (0.5–4.6)	0.43
50–59	5.6 (1.6–19)	0.00*	4.1 (1.1–13.8)	0.04*
≥60 (Ref.)	1		1	
**Ethnicity**				
Punjabi (Ref.)	1		1	
Urdu	2.9 (1.2–7.3)	0.01*	0.6 (0.3–1.4)	0.29
Pushto	1.5 (0.5–4.8)	0.42	0.3 (0.1–0.7)	0.01*
**Occupation**				
Employed (Ref.)	1		1	
Unemployed	6.6 (2.9–14.9)	0.00*	3.6 (1.1–12.2)	0.00*
**Exposure to passive smoking**				
Yes	0.3 (0.1–0.5)	0.00*	0.1 (0.03–0.2)	0.00*
No (Ref.)	1		1	
**Exposure to media**				
None	3.2 (1.8–17.4)	0.00*		
At least one	2.1 (0.7–6.3)	0.17		
All three (Ref.)	1			
**Exposure to health warnings**				
Yes	0.02 (0.01–0.05)	0.00*	0.5 (0.2–1.0)	0.08
No (Ref.)	1		1	
**Intense physical activity**				
Yes (Ref.)	1			
No	1.9 (0.9–4.2)	0.09		

AOR: adjusted odds ratio; adjusted for …..

* Significant at p≤0.05.

## DISCUSSION

Overall, our study found the prevalence of tobacco consumption was 19.1% among poor adults residing in urban slums of Lahore. The finding is similar to a population-based study conducted in Pakistan to assess the prevalence of overall non-communicable related risk factors where tobacco use was 19.7%^14^. The estimated prevalence of tobacco smoking consumption in our study was 10.5% among the adult population, which is lower compared to a nationally representative survey published in Pakistan in 2018 where tobacco use was 39.1%^15^. Similarly, tobacco smoking was reported by 17.1% from the STEPS survey conducted in Nepal and 38.8% in Bangladesh, which is higher than our study findings^16,17^. In Bangladesh, a STEPS survey conducted in the urban slums of Dhaka city showed the proportion of tobacco smokers was 35%, which is quite higher compared to our findings^10^. The estimates in our study are low since tobacco consumption seems to be culturally unacceptable in Pakistani society and social desirability bias might lead to no or less reporting. Additionally, the difference in estimates could be attributed to the different age groups who were interviewed in population-based nationally representative surveys and the WHO STEPS survey (≥18 years) and in different settings.

Our study found that 8.6% of the participants consumed smokeless tobacco among poor urban slum adults, which is very much lower compared to the prevalence from the WHO STEPS survey conducted in urban squatter settlements in Dhaka city, Bangladesh for smokeless tobacco consumption, which was 40.6%^10.^ Whereas, the last Global Adult Tobacco Survey (GATS) survey in 2014 reported consumption of smokeless tobacco consumption was 7.7%, which is similar to our study finding^5^. In recent years, a shift has been observed from smoked to smokeless tobacco use in South-East Asia, where a decline in smoking prevalence by 3% (India), 6% (Bangladesh), and 7% (Nepal) and a rise in smokeless tobacco use increased in India by 6%, Bangladesh by 3%, and Nepal by 4%^18^. Smokeless tobacco consumption might be influenced and encouraged by the policy of banning tobacco smoking introduced in several countries post-FCTC^19^. Further, it has been explored that the use of smokeless tobacco has been perceived by the general population to be less injurious than tobacco smoking in terms of causing cancers and non-communicable diseases, along with nicotine addiction^20^.

Unemployment and no media exposure were found to be important contributing factors, and exposure to health warnings on tobacco packaging to be a protective factor for tobacco product consumption has been found in our study.

The rate of unemployment contributes significantly to increasing the likelihood of tobacco use as anxiety and depression due to the absence of a job could lead a person towards adopting addictive behavior. A study was conducted in China that assessed the relationship between unemployment and smoking and found that a percent increase in the unemployment rate enhanced the likelihood of smoking significantly^21^. Another survey found increased nicotine dependence among smokers who belonged to low socioeconomic and unemployed groups^22^. Employment, working conditions, general health status, education level, and income, strongly influence mental health and are strong contributing factors to tobacco consumption^23,24^.

In our study, participants aged 30–39 years had higher odds of tobacco smoking consumption, which reflects that this age category has more tendency toward tobacco smoking, which might be due to peer pressure, anxiety, or stress due to unemployment or work-related stress. Research studies have shown that all age groups have the tendency to use tobacco products and that tendency increases with ageing. However, the oldest population aged ≥65 years has low tobacco consumption due to multiple health problems^25^.

Unemployment and no media exposure were found to be important contributing factors, and exposure to health warnings on tobacco packaging was found to be a protective factor for tobacco product consumption in our study. The rate of unemployment contributes significantly to increasing the likelihood of tobacco use, as anxiety and depression due to the absence of a job could lead a person to commence an addictive behavior. A Chinese study that examined the relationship between unemployment and smoking found that a percentage increase in unemployment increased the likelihood of smoking significantly^21^. Another survey found increased nicotine dependence among smokers who belonged to low socioeconomic and unemployed groups^22^. Employment, working conditions, general health status, education level, and income strongly influence mental health and are strong contributing factors to tobacco consumption^23,24^. Effective media exposure creates an impact as it spreads awareness on a large scale about the harmful effects of tobacco products. Therefore, it is necessary to implement this targeted intervention among the mass population. The effectiveness of mass media campaigns to reduce tobacco consumption has been evident from different interventional studies which assessed the impact of tobacco control policies’ implementation in terms of mass media campaign coverage, including television advertisements along with emotional messages to encourage quitting the use of tobacco products^26-28^.

Health warning messages on tobacco products’ packaging and labelling on cigarette packs help in quitting tobacco. A trial on cigarette warnings was conducted among smokers ≥18 years, which suggested that labelling protocol and graphic warnings on the front and backside of packaging significantly increased tobacco quitting intentions^29^. The WHO Framework Convention of Tobacco Control (FCTC) has promoted graphic health warning labels (GHWLs) among low- and middle-income countries for the implementation of an effective health warning labels (HWL) policy on tobacco packaging. However, a review found that the weaker states with voluntary HWLs implementation were significantly less likely to be compliant with the policy^30^.

### Policy implications

The high prevalence of tobacco products in our study is of great concern and calls for action, as the use of tobacco products not only increases the disease burden but also contributes to inequities in social and health development^31-33^. The STEPS survey provides the data for policy action that endorse the need to design robust health promotional messages for tobacco cessation^34,35^. The WHO FCTC, since its inception in 2003, has established an approach for international governments to control tobacco^36^. Meanwhile, Pakistan has been a party to the convention since 2004, which aims to devise and implement approaches to decrease tobacco products’ supply and demand by enhancing taxation and stringent regulation^37^.

A multi-pronged approach is required to curb the complex issue of tobacco control with comprehensive and effective implementation of critical policy measures provided by FCTC, which include advocacy and capacity building.

Public education, including mass awareness of the dangers of tobacco use and passive smoking, as well as the placement of health warnings on tobacco packages, may result in behavior change and motivate users to quit smoking sooner rather than later^38,39^. Additionally, the integration of cessation services by healthcare professionals, which includes counselling advice and educating patients on quitting tobacco and treatment for tobacco dependence, into health service delivery at all levels of healthcare, including the community, is crucial^40^. A national quitline for tobacco cessation, pharmacological aids in the form of nicotine replacement therapy, enforcement of smoke-free zones, and policy effectiveness monitoring, should all be in place^41,42^.

### Strengths and limitations

Our study has certain strengths to be considered. This is a community-based, cross-sectional study that shows the representativeness of a sample. Further, an inquiry about tobacco consumption is potentially a sensitive topic, and smoking seemed to be taboo among females, but we could include females with enough participation to provide us with sufficient information. A WHO STEPwise questionnaire, a standardized tool, was used in this study for tobacco consumption assessment. The households were randomly selected within each cluster, which also adds to the strength of the study.

Some limitations need to be considered for this study. There could be a chance of response bias as asking about use, frequency, and type of tobacco might be subject to bias. Additionally, tobacco consumption is self-reported and objective assessment can lead to reporting bias. Further, this is a cross-sectional study, so causality cannot be drawn from it. Moreover, the question of tobacco use by other family members and peers was not included in the STEPS survey; therefore, information on it could not be obtained, which is a potential factor for increasing tobacco use among individuals.

## CONCLUSIONS

This study provides a robust, community-based assessment of tobacco consumption for both smoked and smokeless tobacco. Targeted tobacco prevention strategies are recommended, including the placement of graphical health warnings on packaging, increasing advocacy using a mass media approach, and enhancing counselling efforts for tobacco cessation.

## Data Availability

The data supporting this research are available from the authors on reasonable request.
