# Supplementary material for: Prevalence of tobacco consumption and the associated factors among the adults in an urban slum: Findings from the WHO STEPwise survey
Source: Tob Induc Dis. 2022 Oct 31;90:91. doi: 10.18332/tid/154636 (PMC9619230; doi:10.18332/tid/154636)
Supplement: Supplementary file 1 [file TID-20-91-s1.pdf]

Table S1. Frequency distribution of socio-demographic, exposure related to awareness, life style adopted, health related issues, anthropometric, family history of disease among adults, Lahore, Pakistan (n=607)

**Characteristics**	**Current tobacco Smoking (10.5%) n (%)**		**p-value**	**Current Smokeless Tobacco (8.6%) n (%)**		**p-value**
	**Yes**	**No**		**Yes**	**No**	
Age						
30 to 39 years	18(28.1)	223(41.1)	0.118	25(48.1)	216(38.9)	0.486
40 to 49 years	20(31.1)	127(23.4)		12(23.1)	135(24.3)	
50 to 59	12(18.8)	113(20.8)		7(13.5)	118(21.3)	
≥ 60 years	14(21.9)	80(14.7)		8(15.4)	86(15.5)	
Gender						
Male**	54(84.4)	162(29.8)	0.000	40(76.9)	176(31.7)	0.000
Female	10(15.6)	381(70.2)		12(23.1)	379(68.3)	
Marital Status						
Never married	5(7.8)	17(3.1)	0.058	4(7.7)	18(3.2)	0.101
Ever married	59(92.2)	526(96.9)		48(92.3)	537(96.8)	
Ethnicity *						
Urdu	13(20.3)	216(39.8)		18(34.6)	211(38)	
Punjabi	42(65.6)	251(46.2)	0.006	22(42.3)	271(48.8)	0.142
Pushto	9(14.1)	76(14)		12(23.1)	73(13.2)	
Education						
Intermediate and above	8(12.5)	48(8.8)	0.629	3(5.8)	53(9.5)	0.656
Primary to secondary	26(40.6)	226(41.6)		23(44.2)	229(41.2)	
Illiterate	30(46.9)	269(49.5)		26(50)	273(49.2)	
Socio-economic status						
High income	18(28.1)	136(25)	0.866	10(19.2)	144(25.9)	0.560
Middle income	17(26.6)	150(27.6)		15(28.8)	152(27.4)	
Low income	29(45.3)	257(47.3)		27(51.9)	259(46.7)	
Occupation *						
Employed	49(76.1)	164(30.2)	0.000	37(71.2)	176(31.7)	0.000
Unemployed	15(23.4)	379(69.8)		15(28.8)	379(68.3)	
Exposure to environmental tobacco smoke*						
Yes	35(54.7)	91(16.8)	0.000	38(73.1)	88(15.9)	0.000
No	29(45.3)	452(83.2)		14(26.9)	467(84.1)	
Exposure to Media*						
None	31(48.4)	173(31.9)	0.029	28(53.8)	176(31.7)	0.003
At least one	21(32.8)	228(42)		18(34.6)	231(41.6)	
All three	12(18.2)	142(26.2)		6(11.5)	148(26.7)	
Exposure to health warnings*						
Yes	51(79.7)	68(12.5)	0.000	21(40.4)	98(17.7)	0.000

No	13(20.3)	475(87.5)		31(59.6)	457(82.3)	
Intense Physical Activity*						
Yes	27(42.2)	123(22.7)	0.001	22(42.3)	128(23.1)	0.002
No	37(57.8)	420(77.3)		30(57.7)	427(76.9)	
Moderate Physical Activity						
Yes	18(28.1)	129(23.8)	0.440	13(25)	134(24.1)	0.890
No	46(71.9)	414(76.2)		39(75)	421(75.9)	
Mild Physical Activity						
Yes	34(53.1)	250(46)	0.283	23(44.2)	261(47)	0.699
No	30(46.9)	293(54)		29(55.8)	294(53)	
Have Hypertension*						
Yes	21(32.8)	304(56)	0.000	22(42.3)	303(54.6)	0.089
No	43(67.2)	239(44)		30(57.7)	252(45.4)	
Have Diabetes Mellitus *						
Yes	13(20.3)	177(32.6)	0.045	8(15.4)	182(32.8)	0.010
No	51(79.7)	366(67.4)		44(84.6)	373(67.2)	
Have Hyperlipidemia						
Yes	3(4.7)	66(12.2)	0.075	5(9.6)	64(11.5)	0.677
No	61(95.3)	477(87.8)		47(90.4)	491(88.5)	
Ever advised for quitting tobacco use *						
Yes	29(45.3)	158(29.1)	0.008	23(44.2)	164(29.5)	0.028
No	35(54.7)	385(70.9)		29(55.8)	391(70.5)	
Body Mass Index *						
Normal weight	31(48.4)	159(29.3)	0.005	23(44.2)	167(30.1)	0.095
Overweight	19(29.7)	189(34.8)		16(30.8)	192(34.6)	
Obese	14(21.9)	195(35.9)		13(25)	196(35.3)	
Family History of Stroke						
Yes	3(4.7)	47(8.7)	0.275	7(13.5)	43(7.7)	0.152
No	61(95.3)	496(91.3)		45(86.5)	512(92.3)	
Family History of IHD						
Yes	14(21.9)	96(17.7)	0.410	8(15.4)	102(18.4)	0.592
No	50(78.1)	447(82.3)		44(84.6)	453(81.6)	
Family History of Hypertension						
Yes	21(32.8)	213(39.2)	0.319	25(48.1)	209(37.7)	0.140
No	43(67.2)	330(60.8)		27(51.9)	346(62.3)	
Family History of Diabetes						
Yes	26(40.6)	228(42)	0.834	18(34.6)	236(42.5)	0.269
No	38(59.4)	315(58)		34(65.4)	319(57.5)	

*Significant at $p \leq 0.05$

© 2022 Razzaq S. et al.
